# Re-thinking human interactions with the oceans

**DOI:** 10.1098/rsos.240808

**Published:** 2024-10-02

**Authors:** Michael H. Depledge

**Affiliations:** ^1^ European Centre for Environment and Human Health, University of Exeter Medical School, Peter Lanyon Building, Penryn, Cornwall TR10 8RD, UK

**Keywords:** oceans, human health, wellbeing, pollution

## Abstract

Earth’s marine ecosystems are changing rapidly, in large part owing to the damaging effects of human activities. Unless humans find better ways of interacting with the seas and oceans, the marine resources upon which we rely will diminish as more ecosystems collapse. The consequences for human health and wellbeing will be severe. The meta-discipline of Oceans and Human Health has catalogued how the oceans and their constituents benefit human lives. Examples include access to seafood, pharmaceuticals and physical and mental health benefits. This interdisciplinary research effort has also revealed how the integrated impact of anthropogenic activities has disrupted ocean processes resulting in extensive losses of marine biodiversity, increasing chemical and microbial pollution, proliferation of harmful algal blooms and increased coastal inundation, all of which threaten human populations. In response, non-governmental organizations and national governments have established various agreements and treaties to prevent further damage, restore what has been lost and grasp new economic opportunities. Nevertheless, ocean-related risks continue to escalate rapidly in the absence of political commitment. New thinking regarding the interconnectedness of all human/ocean interactions is required to remove the barriers and impediments that hamper tackling the wicked problem of fostering health and wellbeing while achieving ocean sustainability.

## 1. Background

For thousands of years, humans have been drawn to live close to estuaries, seas and oceans. An estimated 2.15 billion people now live in coastal zones worldwide [1]. By 2030, this is predicted to increase to approximately 5 billion, with growth continuing to populate rapidly expanding coastal towns and cities, primarily in subtropical and tropical regions [2]. This growth trend reflects the panoply of benefits coastal living offers, including access to marine resources, opportunities to trade by sea and a sense of wellbeing that many coastal dwellers derive. Unfortunately, the trend has also resulted in severe damage to marine ecosystems worldwide. Humans have not interacted sustainably with the oceans. The more benefits acquired, the greater the damage, which in turn threatens future benefits.

Studies linking the marine environment and human health have been conducted for centuries. For example, the British physician, Dr Richard Russell (1687–1759), was one of many across Europe who advocated bathing in, and drinking seawater to treat a variety of ills. Another example is the work of Alan Hodgkin and Andrew Huxley at the Plymouth Marine Laboratory during the 1940s, in which squid giant axons were used to investigate the physiology of nerve function. However, it was not until the final decades of the last century, that growing awareness of the extent and complexity of human/ocean interactions prompted a small group of scientists to convene an international symposium in Bermuda in May 1999. They considered an array of anthropogenic impacts on marine life and, at the same time, explored growing threats to human health and wellbeing originating in the oceans [3]. The event contributed to the establishment of the meta-discipline of ‘Oceans and Human Health’ (O&HH), initially in the USA. This interdisciplinary endeavour brought together marine biologists, oceanographers, ecotoxicologists, microbiologists, environmental chemists, medical scientists, epidemiologists, social scientists, environmental psychologists, economists, legal experts and many others in a new venture, in an attempt to overcome the constraints of disciplinary silos to gain new insights into the extensive interconnections between oceans, human health and wellbeing. The United States agencies, including the National Academy of Science, The National Institute of Environmental Health Sciences, the National Science Foundation and the National Oceanographic and Atmospheric Administration, played a vital role in launching and supporting the new meta-discipline. A decade later, the European Union brought together several smaller programmes from across its 27 member states to build its own O&HH programme.

Why oceans should have been singled out for study may not be immediately obvious until their extent and immense size are fully appreciated. Less than one-third of the Earth’s surface is above sea level. The remainder lies submerged beneath seas which contain 97% of all the water on the Earth. Extending through all climatic zones and water depths, the heterogeneous nature of marine ecosystems (including estuaries, coastal wetlands, inshore waters, fjords, lagoons, coral reefs, polar seas, open-oceans and down into the Challenger Deep), is reflected in the diverse influences they have on human populations and cultures around their shores.

The early years of O&HH research focussed on how humans have damaged marine ecosystems, with an implicit assumption that ecological degradation is also detrimental to humans [4]. This led to investigations of the mechanisms by which ocean-related human disease and loss of wellbeing arise [5], and eventually to the equally important topic of the health and wellbeing benefits that we glean from the sea [6]. The meta-discipline of O&HH brings together all that we know so far with the aim of providing the scientific basis for managing human interactions with the oceans more effectively and sustainably in the future. What follows provides a brief overview and a critical assessment of progress.

## 2. What do the oceans do for us?

### 2.1. Direct benefits to health

While only 1% of the living organisms on Earth reside in the oceans, they represent 78% of all animal biomass [7]. Of the 42 extant biological phyla, 34 live in marine ecosystems, represented by more than 238 000 marine species [8]. From this wealth of organisms and their habitats, vital resources are acquired. The extent of our dependence is vividly illustrated by ocean fisheries, which provide approximately 16% of animal protein consumed globally [9]. *Per capita* intake of seafood rose from approximately 10 kg yr^−1^ in the 1960s to approximately 20 kg yr^−1^ in 2022, and continues to rise. Health gains accrue mainly from fish and shellfish protein, and their constituent omega-3 fatty acids which may offer cardio-protective effects, improve neurological function and reduce risks of mammary and prostate cancers [9]. Seaweeds also provide a range of healthy food products. In 2019, more than 34 million tonnes of cultivated production was underway, generating USD 14.7 billion in sales [9,10]. The Food and Agricultural Organization estimates that the livelihoods and wellbeing of over 600 million people depend on all forms of fisheries and aquaculture activities worldwide, of which approximately two-thirds are ocean-based [9].

Many pharmaceuticals have oceanic origins. Marine organism extracts are the basis for several antibiotics and chemotherapeutic agents along with therapeutics for the treatment of pain, cardiovascular disease and eye diseases [10,11]. A notable example is ARA-C (Cytarabine), derived from a marine sponge (*Tectitethya crypta*), and used as a cancer chemotherapy agent to treat leukaemia [11]. To date, 38 000 marine bioactive compounds have been evaluated. Clinical trials of more than 50 new drugs developed from marine organism extracts are currently underway [11].

The oceans also provide other health benefits. In past centuries, visits to the seaside were often employed in helping weak and sickly patients recuperate from illness (so-called thalassotherapy [12]), but with medical advances in the nineteenth century, the practice subsided. In the early 2000s, the ‘Blue Gym’ concept emerged, once again encouraging participation in marine pursuits that promote physical fitness, reduce psychological stress and help to build resilience in mental health [6,13]. By adopting a scientific approach in assessing the value of spending time in and around outdoor blue spaces, significant health benefits were revealed. In England, an analysis of census data for 48 million people showed that they reported better health if they lived at the coast. Effects were especially strong in areas of deprivation, suggesting that coastal living might offer the possibility of helping to address health inequalities [14]. Potential public health benefits include reducing the risk of obesity and of developing diabetes, cardiovascular and respiratory diseases and various cancers [14].

An additional coastal benefit may arise through exposure to biologically active molecules in sea spray. Once inhaled, these agents reduce inflammatory responses and promote better health [15,16], although this has yet to be weighed against potential dangers posed by any toxic constituents in sea spray, such as algal toxins and pollutants.

### 2.2. Indirect benefits through socio-economic development

The relationship between health and wellbeing, and socio-economic conditions is well established; better health is associated with greater employment opportunities and higher incomes [17]. In the context of O&HH, health benefits are generated in this way mainly by trading ocean resources. With the global ocean economy predicted to hit $3 trillion by 2030 [18], a critical factor in sustaining growth will be energy availability. Energy-rich hydrocarbons from the oceans have provided approximately 30% of global oil production and 50% of global natural gas production so far [19]. These fossil fuels have underpinned the transport of goods and people, enabled temperature regulation of homes and workplaces, the manufacture of diverse goods and development of new technologies, as well as many other benefits. To explore how these human needs drive pressures that affect the environment and human health, the European Environment Agency developed the Drivers-Pressures-States-Impacts-Response (DPSIR) framework in 1999. More recently, Reis *et al.* revised this framework to better illustrate the health and social consequences of meeting human needs at the cost of environmental degradation [20]. How these and other frameworks can be used in environmental management has been described by Elliot & Kennish [21].

Less environmentally damaging, renewable energy sources have begun to be employed in supporting human lifestyles in recent years, with the oceans once again playing a key role. Adding to contributions from offshore wind farms, wave and tidal current energy has been captured using point absorbers, barrages and tidal-stream technologies. Other innovative methods are currently under investigation [22]. These energy resources will be essential if an expected 50%–100% increase in global energy requirements is to be met by 2050.

Shipping has also supported improvements in socio-economic development, health and wellbeing. For millennia, fleets of ships have facilitated trade and delivered passengers, as well as essential and non-essential resources, to communities worldwide. In 2020, more than 95% of the volume of world trade involved ships [23], generating jobs in shipbuilding, port operations, logistics and transportation that foster socio-economic improvements. Regarding human health specifically, transportation by sea continues to provide access to essential medical products and services such as food, pharmaceuticals and medical equipment, and aids their distribution to remote and underdeveloped areas [24]. The ocean cruise-ship industry, and indeed maritime tourism more generally, offer socio-economic opportunities for many coastal communities, again bringing health and wellbeing benefits to visitors and local communities alike [25].

Beyond energy provision and transportation, a surprisingly diverse array of business sectors interact with the oceans, ultimately exerting a positive influence on socio-economic conditions, health and wellbeing. The marine aggregates industry, for instance, supplies material for building foundations and drainage systems, for transport infrastructure, for replenishing beaches and in sea defence programmes that protect coastal communities worldwide [26].

## 3. What have we done to the oceans?

Ocean-related health and wellbeing benefits come at an environmental cost. For most of human history, impacts of coastal settlements were inconsequential, reflecting a distributed global population of an estimated 0.5 to 1 billion individuals. However, between 1800 and the present day, the situation has been transformed. The population has grown to over 8 billion, with 40%–50% now residing in maritime regions [27]. The damage inflicted (and which continues to be inflicted) on marine ecosystems, knowingly or inadvertently, is extensive. More than 20 coastal megacities (each with over 10 million inhabitants) have developed along with many smaller coastal cities and towns, leading to the destruction and degradation of coastal and near-shore habitats. Around the Mediterranean Sea for example, more than 40% of the shoreline is covered in man-made structures that have obliterated many seashore habitats [28].

### 3.1. Marine pollution and health

A consequence of locating large populations in maritime areas has been the introduction of a variety of contaminants and pollutants into the oceans. Health threats arise mainly through contamination of seafood. This insidious danger is often seriously underestimated. Persistent chemicals—heavy metals, polyaromatic hydrocarbons (PAH) and a wide range of synthetic organic compounds such as Polychlorinated Biphenyls, bisphenol A, brominated flame retardants, dioxins, perfluorinated compounds, (per and polyfluoro alkyl substances (PFAS)), etc.—found in fish and shellfish have been linked to clinical disorders, including neuropathy, cardiomyopathy, endocrine disruption, vascular disease and cancers [29,30]. Following maternal seafood consumption, foetuses *in utero* and infants have been exposed to methylmercury and PCBs, damaging the developing brain, reducing IQ and increasing children’s risk of autism, attention deficit hyperactivity disorder and learning disorders. Adult exposures to methylmercury increase the risk of cardiovascular disease and dementia. Persistent synthetic chemicals, sometimes bound to ingested microplastics, can disrupt endocrine signalling, reduce male fertility, damage the nervous system and increase cancer risk [30]. Landrigan *et al.* provide many more examples of the routes of uptake of different kinds of marine pollutants [30].

Global chemical production has risen 50-fold since 1950 and is forecast to triple again by 2050, suggesting the likelihood of an ongoing threat [31]. Anthropogenic chemicals in the environment are now almost invariably ingested or absorbed as complex mixtures from marine products and other sources, resulting in elevated body burdens and associated changes in the global incidence and patterns of disease [29].

### 3.2. Wastewater and sewage

Pathogenic microbes discharged with sewage, and with runoff from agricultural activity, pose additional disease threats in both wildlife and humans [32]. There is global concern that wastewater and human sewage of a large portion of the World’s population is still currently discharged, directly or indirectly, into coastal seas, mostly without treatment. What was once simply human excrement is now a complex mixture of toxic waste, including pharmaceutical residues, personal care products and recreational drugs. Research in the late 1990s indicated that sewage pollution generated *ca* 120 million cases of gastrointestinal diseases and more than 50 million cases of respiratory disease annually. Limited availability of recent data obscures current trends, but since the earlier assessments, the global population has increased by 2 billion, mainly in lower and middle-income countries, in subtropical and tropical regions where untreated sewage discharges are most common. By the early 2000s, viral, bacterial and protozoan pathogens polluting coastal waters contaminated edible shellfish and fish resulting in around 4 million cases of hepatitis A and E, which caused 40 000 deaths. A further 40 000 episodes of disease led to long-term disability [32]. With the changing climate and increasing pollution, there is a high risk that *Vibrio* infections, including cholera, will increase in frequency and extend to new areas. This is a particular concern in Asia, Africa and South America, where *Vibrio cholerae* occurs in natural reservoirs in warm coastal waters [33]. The threat of antimicrobial-resistant bacteria in marine ecosystems is also emerging as a critical health consideration around the World [34,35].

### 3.3. Damage associated with offshore oil and gas production

The positive aspects of oil and gas use outlined earlier have to be weighed against the extensive damage resulting from the offshore oil and gas industry globally. Benthic ecosystems in particular, have been subjected to severe physical disturbance, chemical pollution and noise pollution, contributing to the decline of fisheries and reducing the amenity value of coastal areas [36]. Toxic chemicals from drilling processes contaminate seafood while accidents, such as major oil spills, result in long-term effects on the health of oil industry workers and nearby coastal communities. A notable example was the Deepwater Horizon drilling rig incident in 2010. Litchveld *et al.* claim that up to 210 million gallons of crude oil were released into the Gulf of Mexico, along with 1.8 million gallons of chemical dispersant, severely affecting 40% of all the coastal wetland in the USA. As a result, seafood businesses involving shrimp, lobster, oysters, juvenile fishes and seaweed declined. Oil workers, rescuers and coastal inhabitants were exposed to air polluted with carcinogenic PAHs and volatile organic compounds released from dispersants. Heavy metal exposures also occurred, most notably in children [37]. However, it is important to note that further in-depth investigations revealed a more complex picture. To fully assess the impact of the Deepwater Horizon spill the quantities of materials that entered the ocean, along with the amounts of dispersants used during clean-up, needed to be determined. The chemical composition of the released materials and their relative proportions was also critically important [38]. Integrated modelling of the long-term impacts of such incidents may offer a better way of providing understanding of the damage caused. For example, in the case of Deepwater Horizon, negative impacts on seafood businesses and tourism were related to some extent to the perceived, rather than actual risk of seafood contamination, driven by media reports [39].

Incidents continue to occur around the World, and are reminders of past, highly damaging, marine accidents (for instance, those involving the oil tankers Amoco Cadiz, Atlantic Empress, Castillo de Belver, Torrey Canyon and Exxon Valdez).

### 3.4. Potentiation by climate change

Impacts of anthropogenic climate change, related in part to the burning of marine-derived fossil fuels, have been documented in numerous Intergovernmental Panel on Climate Change reports and will not be reiterated here (see [40]). The resulting global warming not only poses a major threat to human health and wellbeing, but also has already severely disrupted and degraded marine ecosystems worldwide, leading to the collapse of some [41]. More than 90% of the heat generated through greenhouse warming resides in the sea, causing extensive damage such as the bleaching of coral reefs and the melting of polar ice [40]. It is widely recognized that climate change potentiates other threats by increasing the frequency and intensity of storms, driving sea level rise and coastal flooding, exacerbating biodiversity loss and damaging health [40]. Ocean acidification, caused by atmospheric carbon dioxide dissolving in the oceans, is another human health risk in the long term [42]. As the climate changes, physico-chemical conditions in the seas are being transformed, driving the redistribution of species and pollutants, altering the latter’s bioavailability and toxicity and leading to further disruption of fisheries, tourism, lives and livelihoods.

An ever-changing picture of climate-ocean effects is now widely acknowledged, along with increasing health risks everywhere [40]. The challenge remains to find ways of using all types of marine resources without destroying the source of the resource. Even a readily manageable activity such as commercial fishing, which delivers essential food for humans worldwide, has become one of the most disruptive activities in the marine environment, leading to continuing ecosystem degradation, with half of the World’s fish stocks being overfished [43].

### 3.5. Interactions between anthropogenic and natural threats

Not all threats arising in the ocean are owing to human activities. A variety of naturally occurring phenomena interact with anthropogenic disturbance. For example, the Asian earthquake and tsunami of December 2004 killed at least 226 000 people in the coastal areas of 13 countries, with over 500 000 people injured, often with long-term health consequences. A further 150 000 people died from infectious diseases following the disaster [44]. The removal of coastal protection by the destruction of mangroves undoubtedly magnified the impact of the tsunami in some locations.

Harmful algal blooms (HABs) develop naturally when particular algal species proliferate as nutrient availability changes, although other factors influence their development too, such as anthropogenic pollution and climate change [45]. Blooms in coastal waters often disrupt local fisheries, tourism and the local economy. By the early 2000s, roughly 60 000 cases of gastroenteritis and respiratory irritation arose annually owing to consumption of seafood containing algal toxins [32,44]. Only a small proportion were diagnosed, predominantly as amnesic shellfish poisoning, paralytic shellfish poisoning, diarrhetic shellfish poisoning, neurotoxic shellfish poisoning and ciguatera poisoning [32,45]. Consequences may include severe neurological impairment and rapid death. Also, HAB toxins can become airborne in sea spray causing respiratory disease [30]. Sufficient data are not currently available to assess the extent of the threat globally.

### 3.6. Human behaviour and activities

Risks to health are strongly influenced by the activities humans engage in while in marine environments. Of note, over 200 000 people drown in the sea each year. Those working in fisheries and the offshore oil industry are especially at risk. More than 355 000 people per annum are injured globally in recreational boating accidents, with 40% of these injuries requiring significant medical treatment. Recreation in coastal waters can result in exposure to dangerous marine organisms, including some invasive species [44], along with the pathogenic microbes and algal toxins mentioned earlier.

## 4. Why are we failing to protect the oceans and human health?

Oceans and human health research shows that despite the array of measures put forward to prevent further degradation and mitigate numerous serious health risks, the beneficial aspects of human interactions with the oceans are being undermined. What then are the barriers to more effective action?

### 4.1. Prevarication and the precautionary principle

Slow responses to O&HH issues have allowed the insidious development of multiple threats. Two illustrative examples from an extensive list are the presence of ‘forever chemicals’ (PFAS) and the emergence of antimicrobial resistance in marine ecosystems. To emphasize how long it takes to build a sufficient weight-of-evidence before responsible bodies begin to address a potential threat, it is noteworthy that PFAS were dubbed ‘forever chemicals’ by the United States Environmental Protection Agency back in 1998 owing to their persistence and toxicity; yet it has taken more than a quarter of a century to begin to limit their release into the oceans. Regarding antimicrobial resistance (AMR), data gathering in marine ecosystems has only recently begun in earnest, even though the potential for significant widespread harm to humans through exposure to seafood contaminated with antibiotic-resistant microbes, or while taking holidays on AMR-contaminated beaches, has been known for more than a decade [34,35].

The Precautionary Principle was proposed in 1992 to ensure that safety measures are applied early enough to prevent or mitigate such risks [46]. It states that *‘The absence of certainties……must not delay the adoption of effective and proportionate preventative measures aimed at forestalling a risk of grave and irreversible damage to the environment, at an economically calculable cost’* [47]. Often this has not been achieved. A report by Gee *et al.* entitled ‘Late lessons from early warnings’ described 12 case studies in which information was available at an early stage about particular environmental or health threats that should have led to immediate responses [48]. Instead, evidence was ignored until severe damage occurred. In 2013, Gee *et al.* extended their studies, adding to the list of environmental threats that had not elicited appropriate precautionary responses [49], but again no noticeable change in the approach of responsible bodies followed.

Numerous real-world cases have confirmed retrospectively that when the weight of accumulating evidence points to a potential danger, it is wise to be cautious, even if available data at the time did not clearly reveal the precise mechanism or extent of the threat [50].

Plastics are a case in point. The potential threat offered by marine plastic litter (including very small plastic particles) was first noted during the 1960s and 1970s, and highlighted again throughout the 1980s [36] and early 2000s [4]. Warnings went unheeded until the small plastic particles eventually gained media attention after being dubbed ‘microplastics’ [51]. Nonetheless, quantities entering the oceans are still expected to treble within the decade [18]. Ten years ago, studies demonstrated that both wildlife and humans were being exposed to microplastics (and the chemicals and microbes that are transported on the particles), potentially posing an increased risk of disease [52]. However, health threats were put aside by responsible bodies until fully proven [52]. Since then, more toxicity studies have been completed indicating that microplastics are indeed damaging wildlife and human health, having been found in blood, lungs, breast milk, semen and placenta, and in association with human cell damage, declining sperm counts and irritable bowel syndrome [53,54]. These examples not only highlight the danger of failing to adopt a weight-of-evidence approach and implement the precautionary principle, but also the continuing inherent weakness of current evaluation and regulatory procedures.

### 4.2. Limited effectiveness of international initiatives

The overwhelming evidence of degradation and damage in our seas and oceans led the United Nations to declare the period 2021 to 2030, the Decade for Ocean Science for Sustainable Development—the ‘Ocean Decade’ [55], with the aim of achieving:

- a clean ocean where sources of pollution are identified and reduced or removed;
- a healthy and resilient ocean where marine ecosystems are understood, protected, restored and managed;
- a productive ocean supporting a sustainable food supply and a sustainable ocean economy;
- a predicted ocean where society understands and can respond to changing ocean conditions;
- a safe ocean where life and livelihoods are protected from ocean-related hazards;
- an accessible ocean with open and equitable access to data, information and technology and innovation; and
- an inspiring and engaging ocean where society understands and values the ocean in relation to human wellbeing and sustainable development [55].

This welcome attempt to set a course for managing the oceans is not the first to voice such aspirations. The inspirational marine biologist Rachel Carson, author of the seminal text ‘Silent Spring’, warned in her earlier book, ‘The Sea Around Us’ published in 1951, of the warming oceans and of the adverse impacts that marine pollution was having on human lives [56]. Forty-one years after her call to action and following the signing of numerous international agreements and conventions, (such as the International Convention for prevention of pollution of the sea by oil (1954), the London Convention (1972), the International Convention for Prevention of Pollution from Ships - MARPOL (1973), the United Nations Convention on the Law of the Sea (UNCLOS, 1982), the World Ocean Assessments (Intergovernmental Oceanographic Commission (IOC), 2015, 2021), regional seas conventions (e.g. Oslo-Paris Convention, Helsinki Convention) and pollution control efforts such as the Mediterranean Action Plan-MEDPOL (1975)), a major push was initiated by the United Nations to highlight the severity of the ocean’s plight and its implications for the global community at its Agenda 21 Earth Summit in 1992 [57]. The hope was that policies proposed would finally lead to protection of the seas by the start of the twenty-first century. Further national and international attempts to track progress followed, including the Health of the Oceans (HOTO) report of 2002 [4], sponsored by three leading international bodies, the IOC, the International Maritime Organization and the United Nations Environment Programme. HOTO’s authors performed an in-depth analysis of the state of the oceans, pointing out that the primary concerns they were addressing were the same as had been identified 10 years earlier in Agenda 21, namely habitat loss, changes in phytoplankton abundance and diversity, collapse of fisheries, aquatic toxins, excess nutrients, petroleum hydrocarbons (oil and gas), artificial radionuclides, pharmaceuticals, dissolved oxygen, herbicides, pesticides, biocides, suspended particulate matter, human pathogens, synthetic organic chemicals/persistent organic pollutants, litter, plastics, polycyclic aromatic hydrocarbons (PAHs), metals and organometals [4]. By 2015, the United Nations Member States realized they needed to further invigorate their efforts by adopting 17 Sustainable Development Goals (SDGs) to be achieved by 2030 [58]. SDG no. 3 and no. 14 are most relevant to O&HH, dealing with ‘health and wellbeing’, and ‘conserving and sustainably using marine resources’, respectively. Despite this commitment, Landrigan *et al.* again concluded in 2020 that the critical issues from 1992 remained unresolved [30]. Indeed, the situation had actually deteriorated further as the list of chemical contaminants had increased and plastics of all types and sizes littered seashores and seas globally.

## 5. Re-thinking oceans and human health policies

### 5.1. Wicked problems

**Figure 1.**
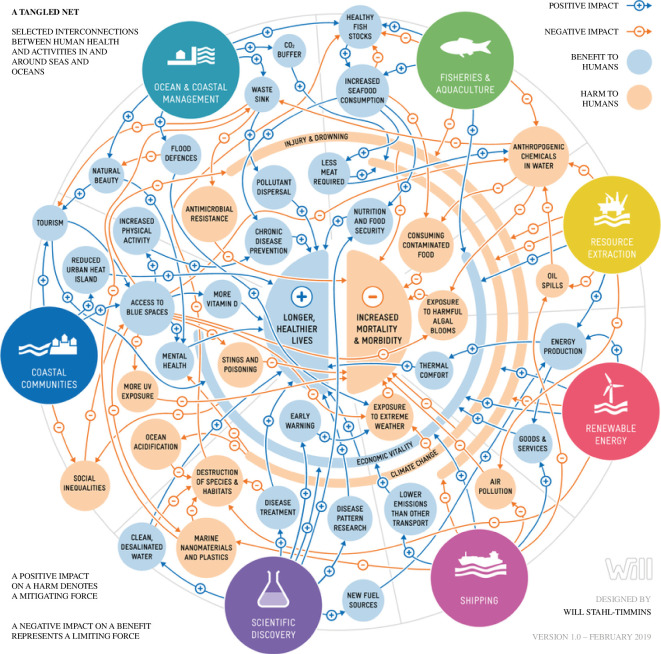
The matrix of Oceans and Human Health connections.

Policymakers have tended to treat as separate issues the protection of the oceans and the human health threats related to the ocean. Within these two domains, topics such as overfishing, coastal flooding, biodiversity loss and mental health in coastal communities are also treated separately. However, findings from across the O&HH meta-discipline show that many marine topics are intimately interconnected and better tackled together. This necessitates advice being sought from a diverse range of experts, drawn from academia, research institutes, government agencies, the business community and a variety of non-governmental organizations. The public at large should also be included, particularly those from coastal communities where exposure to health hazards and benefits received from the ocean can be a daily occurrence. In this way, a broad suite of evidence can be assembled upon which to base policy recommendations. An extraordinarily complex matrix of ocean and human health connections becomes apparent as shown in figure 1 [59,60].

Challenges of this nature have been designated ‘wicked problems’. They are characterized by being poorly defined, with incomplete information and complex changing circumstances that render decision-making extremely difficult [61]. Addressing one part of the problem may initiate unintended consequences elsewhere. A suite of actions addressing multiple facets of the wicked problem is usually required to improve the direction of travel to a more sustainable, beneficial trajectory. Early identification of newly emerging issues is also vital, for which horizon scanning has proved to be an invaluable tool [62].

### 5.2. Human health considerations in ocean protection policies

The United Nations Decade of the Ocean’s Panel launched a so-called Blue Paper at their meeting in Barcelona in April 2024 [63]. The paper states that ‘a healthy ocean is imperative for human health, that oceans offer unrecognized and unrealized potential to improve human health, can support mental health and wellbeing, create economic opportunity and advance social justice’. This proposed shift of emphasis to include human health and wellbeing alongside the state of the oceans marks an important change in thinking, strongly reflecting the findings of the O&HH research community. The paper goes on to repeat calls for urgent and comprehensive action to restore marine ecosystems, although in this regard, differs very little from what was proposed within Agenda 21 in 1992. It remains clear what needs to be done, but not always why it is not being done.

A newly modified policy that has been progressively adopted, albeit inconsistently, is the creation of marine protected areas (MPAs) together with other effective conservation measures, to exclude at least some of the factors that may damage marine ecosystems and restore their proper functioning [64]. Although generating some local resistance from those who perceive a threat to their livelihoods, the cost of political backing for such measures has usually been found to be acceptable. Enforcement of the rules concerning operation of MPAs does, however, need strengthening for which the support of coastal communities is essential. Pointing out the health and socio-economic benefits of restoring marine ecosystems is an important first step [20,64].

A related policy initiative that has also garnered support from both governmental and non-governmental organizations is the ‘30 × 30’ nature conservation target agreed at COP15 in Montreal in December 2022. One of the key objectives is to ensure that 30% of the World’s oceans receive protected status by 2030 [65]. On land, many natural ecosystems have been modified to meet human needs (for example, by farming, forestry and development of urban environments). A debate is required to discuss analogous cases in marine ecosystems. Coastal areas have already been modified for shipping, tourism, leisure pursuits, windfarms, mariculture, land development and reclamation and oil and gas exploration, but without much thought as to how such activities fit together or whether the public considers these developments desirable. Planning more openly and in greater depth for the longer term is likely to achieve better outcomes for the oceans and human health.

### 5.3. Intensification of protective measures to avoid harm

Restricting the disruption of coastal and offshore marine habitats, including that caused by mining the deep sea, markedly reducing the release of chemicals into the oceans, minimizing the discharge of untreated sewage and microbial pollutants, curtailing agricultural runoff, minimizing light and sound pollution and making sustainable fishing and mariculture practices mandatory, are key proposals that have been advocated by international bodies and some governments. All of this is necessary while reducing greenhouse gas emissions to combat global warming, which in turn will help to address sea level rise and ocean acidification.

Some of the above measures are already in place somewhere in the World, but by no means everywhere. It is particularly concerning that the intensity with which they are applied is still insufficient to halt decline. This was recognized by Collins *et al.* [31]. They proposed that among the best practical actions to reduce pollution would be to stop the production of persistent contaminants, facilitate a major step change in recycling, promote green chemistry (which aims to reduce or eliminate the generation of hazardous substances) and change specific environmental management practices in agriculture and industry to minimize the release of contaminants. However, their overriding recommendation was that a truly enormous intensification of all of these activities should take place [31].

### 5.4. Involving coastal communities

Eighty-five per cent of the World’s *ca* 1.6 million kilometres of coastline have been disturbed by human enterprises [66,67]. Even so, many of those living in coastal towns and villages around the World still experience a lack of economic opportunities and under-provision of health services for their ageing populations. Local industries based around fishing, port work or tourism often offer only part-time or seasonal work. Such demographic factors promote inequality and disease conditions [68,69]. Severe storms, sea-level rise and coastal flooding add to the challenges, especially for coastal communities in subtropical and tropical, lower and middle-income countries. Those living in urbanized, low-lying, poorly developed areas and on oceanic islands are especially at risk. It is alarming that at a time when climate change is greatly exacerbating these threats, more people, through choice or necessity, are moving to live close to, or at, the sea coast. Making progress in protecting coastal communities requires more effective outreach and education programmes to raise ocean literacy and awareness of the ocean-related benefits and risks for society and for personal health and wellbeing.

### 5.5. Re-thinking our approaches

Accepting that humans are failing to live sustainably with the oceans is the first challenge to be overcome. Next, we need to decide what to do differently in the future. In the previous sections, some of the persistent problems are outlined as well as ways to address them.

However, this is unlikely to be sufficient alone. Excessively complex policies and failure to implement them properly are hallmarks of weak, ill-informed governments. Greater efforts to educate elected representatives about ocean issues are essential. In the UK, members of the scientific community and non-governmental organizations have called for the appointment of a Minister for the Oceans to constantly make the case for taking greater care of the marine environment while exploring new opportunities to foster human health and wellbeing. At present this remains an ambition, but pressing for the appointment of a high-ranking government advisor, (a so-called Ocean Czar), to keep oceans on the political and public agenda, may be within reach. Chief scientific advisors to government ministers and their various departments, supported by their country’s science academies, should also become more engaged and proactive in driving ministers and senior civil servants to take effective actions in the areas for which their responsibilities impinge on ocean health.

The experience in the USA in the early days of the O&HH meta-discipline, when several funding bodies and agencies worked together to foster O&HH research, is one that should be replicated, at least to some degree, in countries around the World, especially in those which have currently failed to take their responsibilities seriously. Deep frustration with the lack of effective action on environmental and health issues eventually gives rise to public protests and campaigns. With regard to the oceans, this is ultimately likely to emerge more forcibly than is apparent today. Hopefully, intensive efforts to increase ocean literacy in all sections of society will create sufficient impetus to make it clear to responsible bodies that they must do a great deal better when it comes to protecting the O&HH. Failure is not an option.

## 6. Summary

The influence of the state of the World’s oceans on human health and wellbeing has been underestimated for decades, if not centuries. Despite numerous national and international efforts to protect marine ecosystems they continue to degrade. Over-exploitation of marine resources, the physical destruction of habitats and the careless release of pollutants threaten the plethora of socio-economic and health benefits from the oceans upon which we have come to rely. If remedial action is not taken, collapsing marine ecosystems, under additional pressures from climate change, will pose ever greater risks to human lives [41]. Among the main barriers to more effective action are prevarication and delay when threats emerge, failure to invoke the precautionary principle in the face of economic pressures, weak and ineffective assessment of old and new threats, timid scientific advisory committees who fail to urge caution, failure to produce policies that escalate and intensify measures needed to halt further damage, weak governments who ignore their responsibilities and a general lack of awareness among politicians, policymakers and the wider public of the extent of our dependence on healthy oceans in the short, medium and long term.

In moving forward, far greater attention must be paid to the health and wellbeing of coastal communities and to the needs of those elsewhere who use marine resources from time to time, or in some way rely on the marine environment for their livelihoods or wellbeing. The O&HH meta-discipline has led to a growing appreciation of the intimate interconnections between the state of marine ecosystems and human health. Despite the aspirations expressed in the succession of ocean-related conferences and international agreements to protect and restore marine ecosystems, and to take advantage of the benefits they offer, little is likely to change unless politicians and policymakers recognize their duties and show the necessary political commitment to ensure that they themselves, along with coastal communities and the broader public, become involved in deciding how to live responsibly, healthily and sustainably with the oceans in future. The global population has tripled since Rachel Carson and others raised concerns about human impacts on the seas and oceans almost 75 years ago. Anthropogenic pressures will increase further still in the coming decades. Unless our thinking changes, by the time the population triples again, the oceans may cease to exist as we know them today.

## Data Availability

This article has no additional data.

## References

[1] Reimann L, Vafeidis AT, Honsel LE. 2023 Population development as a driver of coastal risk: current trends and future pathways. Camb. Prisms Coast. Futures **1**, 1–12. (10.1017/cft.2023.3)

[2] Tibbetts J. 2002 Coastal cities: living on the edge. Environ. Health Perspect. **110**, A674–81. (10.1289/ehp.110-a674) 12417494 PMC1241085

[3] Depledge MH, Harvey AJ, Brownlee C, Frost M, Moore MN, Fleming LE. 2013 Changing views of the interconnections between the oceans and human health in europe. Microb. Ecol. **65**, 852–859. (10.1007/s00248-012-0173-0) 23325465

[4] HOTO. 2002 The final plan for the health of the oceans module of GOOS. GOOS, 99 IOC/INF-1167. UNESCO.

[5] Bowen R, Depledge M, Carlarne C, Fleming LE. 2014 Oceans and human health: implications for society and well-being. Chichester, UK: John Wiley & Sons Ltd.

[6] Depledge MH, Bird WJ. 2009 The blue gym: health and wellbeing from our coasts. Mar. Pollut. Bull. **58**, 947–948. (10.1016/j.marpolbul.2009.04.019) 19476955

[7] Ritchie H. 2019 Humans make up just 0.01% of Earth’s life – whats the rest? See https://ourworldindata.org/life-on-earth.

[8] Katona S, Paulikas D, Ali S, Clarke M, Ilves E, Lovejoy TE, Madin LP, Stone GS. 2023 Land and deep-sea mining: the challenges of comparing biodiversity impacts. Biodivers. Conserv. **32**, 1125–1164. (10.1007/s10531-023-02558-2)

[9] FAO. 2022 Food and agricultural organisation of the united nations. The state of world fisheries and aquaculture. See https://www.fao.org/home/en.

[10] Douzmeizel V. 2022 The seaweed revolution, p. 279. London, UK: Hero, Legend Times Group Ltd.

[11] Antunes EM, Beukes DR, Caro-Diaz EJE, Narchi NE, Tan LT, Gerwick WH. 2023 Medicines from the sea. In Oceans and human health: opportunities and impacts, 2nd edn (eds LE Fleming, LB Alcantara Creencia, WH Gerwick, HC Goh, MO Gribble, B Maycock, H Solo-Gabriele), pp. 103–148. London, UK: Academic Press.

[12] Lucchetta MC, Monaco G, Valenzi VI, Russo MV. 2013 The historical-scientific bases of thalassotherapy: state of the art. Clin. Ter. **158**, 533–541. 18265720

[13] White MP, Bell S, Jenkin R, Wheeler B, Depledge M. 2016 Green exercise. In Linking nature, health and well-being (eds J Barton, R Bragg, C Wood, J Pretty), pp. 69–78. London, UK: Routledge.

[14] Wheeler BW, White M, Stahl-Timmins W, Depledge MH. 2012 Does living by the coast improve health and wellbeing? Health Place **18**, 1198–1201. (10.1016/j.healthplace.2012.06.015) 22796370

[15] Moore MN. 2015 Do airborne biogenic chemicals interact with the PI3K/akt/mtor cell signalling pathway to benefit human health and wellbeing in rural and coastal environments? Environ. Res. **140**, 65–75. (10.1016/j.envres.2015.03.015) 25825132

[16] Asselman J, Van Acker E, De Rijcke M, Tilleman L, Van Nieuwerburgh F, Mees J, De Schamphelaere KAC, Janssen CR. 2019 Marine biogenics in sea spray aerosols interact with the MTOR signaling pathway. Sci. Rep. **9**. (10.1038/s41598-018-36866-3) PMC6345880 30679557

[17] WHO. 2024 Social determinants of health. See https://www.who.int/.

[18] Government Office of Science. 2018 Foresight: future of the sea. See https://assets.publishing.service.gov.uk/government/uploads/system/uploads/attachment_data/file/639432/Health_and_Wellbeing_Final.pdf.

[19] NOAA. 2023 Energy from the oceans. See https://www.noaa.gov/.

[20] Reis S *et al*. 2015 Integrating health and environmental impact analysis. Public Health **129**, 1383–1389. (10.1016/j.puhe.2013.07.006) 24099716

[21] Elliot M, Kennish MJ. 2024 A synthesis of anthropogenic impacts and solutions in estuarine and coastal environment. In Anthropogenic uses, effects, and solutions on estuarine and coastal systems 6 of the treatise on estuarine and coastal science, 2nd edn (eds MJ Kennish, M Elliott), pp. 1–56. London, UK: Elsevier. (10.1016/B978-0-323-90798-9.00126-8)

[22] IRENA. 2022 Scaling up investments in ocean energy technologies. See https://www.irena.org/irena.org.

[23] UNCTAD. 2020 UNCTAD annual report. See https://unctad.org/statistics.

[24] ITF. 2017 Quantifying the socio-economic benefits of transport, ITF Roundtable Reports. Paris, France: OECD Publishing. See 10.1787/9789282108093-en.

[25] Smith TF, Elrick-Barr CE, Thomsen DC, Celliers L, Le Tissier M. 2023 Impacts of tourism on coastal areas. Camb. Prisms Coast. Futures **1**, e5. (10.1017/cft.2022.5)

[26] Crown Estate. 2023 Marine. See https://www.thecrownestate.co.uk/.

[27] Roser M, Ritchie H. 2023 How has world population growth changed over time? See https://ourworldindata.org/.

[28] UNEP. 2018 UNEP report. See https://www.unep.org/unepmap/.

[29] Depledge MH, Tyrrell J, Fleming LE, Holgate ST. 2013 Are marine environmental pollutants influencing global patterns of human disease? Mar. Environ. Res. **83**, 93–95. (10.1016/j.marenvres.2012.10.003) 23140902

[30] Landrigan PJ *et al*. 2020 Human health and ocean pollution. Ann. Glob. Health. Dec. **86**, 151. (10.5334/aogh.2831) PMC7731724 33354517

[31] Collins C, Depledge M, Fraser R, Johnson A, Hutchison G, Matthiessen P, Murphy R, Owens S, Sumpter J. 2020 Key actions for a sustainable chemicals policy. Environ. Int. **137**, 105463. (10.1016/j.envint.2020.105463) 32086074

[32] Shuval H. 2003 Estimating the global burden of thalassogenic diseases: human infectious diseases caused by wastewater pollution of the marine environment. J. Water Health **1**, 53–64. 15382734

[33] CDC. 2022 Centre for disease control global annual report. CDC advances health equity around the world. See https://www.cdc.gov/.

[34] Gaze W, Depledge M. 2017 Antimicrobial resistance: investigating environmental dimensions. In Frontiers: emerging issues of environmental concern, united nations environment programme, pp. 12–22. Paris, France: Frontiers, UNEP. See https://www.unep.org/.

[35] Leonard AFC, Singer A, Ukoumunne OC, Gaze WH, Garside R. 2018 Is it safe to go back into the water? A systematic review and meta-analysis of the risk of acquiring infections from recreational exposure to seawater. Int. J. Epidemiol. **47**, 572–586. (10.1093/ije/dyx281) 29529201 PMC5913622

[36] Clark RB. 1986 Marine pollution. Oxford, UK: Oxford Science Publications.

[37] Lichtveld M, Sherchan S, Gam KB, Kwok RK, Mundorf C, Shankar A, Soares L. 2016 The Deepwater Horizon oil spill through the lens of human health and the ecosystem. Curr. Environ. Health Rep. **3**, 370–378. (10.1007/s40572-016-0119-7) 27722880 PMC5112119

[38] Rullkotter J, Farrington JW. 2012 What was released? Oceanography **34**, 44–55. (10.5670/oceanog.2021.116)

[39] Solo-Gabriele S *et al*. 2021 Towards integrated modeling of the long-term impacts of oil spills. Mar. Policy **131**, 104554. https://www.sciencedirect.com/10.1016/j.marpol.2021.104554 PMC10581399 37850151

[40] IPCC. 2023 Intergovernmental panel on climate change AR synthesis report: climate change. (10.1017/9781009157988). See https://www.ipcc.ch/.

[41] Bergstrom DM *et al*. 2021 Combating ecosystem collapse from the tropics to the antarctic. Glob. Chang. Biol. **27**, 1692–1703. (10.1111/gcb.15539) 33629799

[42] Patz JA, Olson SH, Gray AL. 2006 Climate change, oceans, and human health. Oceanography **19**, 52–59. (10.5670/oceanog.2006.64)

[43] UNFCCC. 2022 UN Climate Change Annual Report 2022 (unfccc.int). See https://unfccc.int/.

[44] Depledge MH *et al*. 2018 Oceans, health and wellbeing. In The future of ocean governance and capacity development: essays in honor of elisabeth mann borgese (ed. D Werle), pp. 99–204. Leiden, Netherlands: Brill Nijhoff. (10.1163/9789004380271_034). See http://www.jstor.org/stable/10.1163/j.ctv2gjwvhb.

[45] Berdalet E, Chinain M, Kirkpatrick B, Tester PA. 2023 Harmful algal blooms cause ocean illnesses affecting human health. In Oceans and human health, opportunities and impacts (eds LB Fleming, WH Gerwick, HC Goh, MO Gribble, H Solo-Gabriele), pp. 289–314, 2nd edn. London, UK: Academic Press. (10.1016/B978-0-323-95227-9.00020-8)

[46] Kriebel D *et al*. 2001 The precautionary principle in environmental science. Environ. Health Perspect. **109**, 871–876. (10.1289/ehp.01109871) PMC1240435 11673114

[47] Gollier C, Treich N. 2003 Decision-making under scientific uncertainty: the economics of the precautionary principle. J. Risk Uncertainty **27**, 81.

[48] EEA. 2001 Late lessons from early warnings: the precautionary principle. Copenhagen, Denmark: European Environment Agency. See https://european-union.europa.eu/index_en.

[49] EEA. 2013 Late lessons from early warnings: science, precaution and innovation. Copenhagen, Denmark: European Environment Agency. See https://european-union.europa.eu/index_en.

[50] Johnson AC, Sumpter JP, Depledge MH. 2021 The future of the weight-of-evidence approach: a response to Suter’s comments. Environ. Toxicol. Chem. **40**, 2947–2949. (10.1002/etc.5215) 34699630 PMC9298366

[51] Thompson RC, Olsen Y, Mitchell RP, Davis A, Rowland SJ, John AWG, McGonigle D, Russell AE. 2004 Lost at sea: where is all the plastic? Science **304**, 838. (10.1126/science.1094559) 15131299

[52] Leslie HA, Depledge MH. 2020 Where is the evidence that human exposure to microplastics is safe? Environ. Int. **142**, 105807. (10.1016/j.envint.2020.105807) 32599356 PMC7319653

[53] Danopoulos E, Twiddy M, West R, Rotchell JM. 2022 A rapid review and meta-regression analyses of the toxicological impacts of microplastic exposure in human cells. J. Hazard. Mater. **427**, 127861. (10.1016/j.jhazmat.2021.127861) 34863566

[54] Garcia MA *et al*. 2024 Quantitation and identification of microplastics accumulation in human placental specimens using pyrolysis gas chromatography mass spectrometry. Toxicol. Sci. **199**, 81–88. (10.1093/toxsci/kfae021) 38366932 PMC11057519

[55] UN Ocean Decade. 2021 United Nations decade of ocean science for sustainable development. See https://www.unesco.org/en.

[56] Carson RL. 1951 The sea around us. Oxford, UK: Oxford University Press.

[57] Department of Economic and Social Affairs. 1992 Agenda 21. Protection of the oceans, all kinds of seas, including enclosed and semi-enclosed seas, and coastalareas and the protection, rational use and development of their living resourcespages 1–136. https://www.un.org/

[58] UNDP. 2015 Sustainable Development Goals – United Nations. Department of Economic and Social Affairs. See https://www.un.org/.

[59] Fleming LE, Maycock B, White MP, Depledge MH. 2019 Fostering human health through ocean sustainability in the 21st century. People and Nat. **1**, 276–283. (10.1002/pan3.10038)

[60] Depledge MH, White MP, Maycock B, Fleming LE. 2019 Time and tide: our future health depends on the oceans. Brit. Med. J. **366**, l4671. (10.1136/bmj.l4671) 31315830 PMC6635814

[61] Churchman CW. 1967 Wicked problems. Managmt. Sci. **14**, B141–B142.

[62] Herbert-Read JE *et al*. 2022 A global horizon scan of issues impacting marine and coastal biodiversity conservation. Nat. Ecol. Evol. **6**, 1262–1270. (10.1038/s41559-022-01812-0) 35798839

[63] Fleming LE *et al*. 2024 How can a healthy ocean improve human health and enhance wellbeing on a rapidly changing planet? Washington, DC: World Resources Institute. See https://eur03.safelinks.protection.outlook.com/?url=https%3A%2F%2Foceanpanel.org%2Fpublications%2Focean-human-health%2F&data=05%7C02%7CM.Depledge%40exeter.ac.uk%7Cec12af5f77d24d1bf0b108dc604b1c83%7C912a5d77fb984eeeaf321334d8f04a53%7C0%7C0%7C638491123065948043%7CUnknown%7CTWFpbGZsb3d8eyJWIjoiMC4wLjAwMDAiLCJQIjoiV2luMzIiLCJBTiI6Ik1haWwiLCJXVCI6Mn0%3D%7C0%7C%7C%7C&sdata=Fvcwq93BGInRRPG9MKBM4beIjHStaC1FXcwBlIDC23w%3D&reserved=0.

[64] Ban NC *et al*. 2019 Well-being outcomes of marine protected areas. Nat. Sustain. **2**, 524–532. (10.1038/s41893-019-0306-2)

[65] Natural England. 2023 30 by 30: a boost for nature recovery.. See https://www.blog.gov.uk/.

[66] World Resources Institute. 2001 Coastal ecosystems. See https://www.wri.org/.

[67] WEF. 2022 Only 15% of the world’s coastlines remain in their natural state. World Economic Forum. See https://www.weforum.org/.

[68] Depledge MH, Lovell R, Wheeler BW, Morrissey KM, White MP, Fleming LE. 2017 Future of the sea: health and well‐being of coastal communities. UK: government office for science. See https://assets.publishing.service.gov.uk/government/uploads/system/uploads/attachment_data/file/639432/Health_and_Wellbeing_Final.pdf.

[69] CMO. 2021 Health in coastal communities – summary and recommendations. See https://www.gov.uk/government/publications/chief-medical-officers-annual-report-2021-health-in-coastal-communities.

